# Effect of Probiotic Supplementation on Cognitive Function and Metabolic Status in Alzheimer's Disease: A Randomized, Double-Blind and Controlled Trial

**DOI:** 10.3389/fnagi.2016.00256

**Published:** 2016-11-10

**Authors:** Elmira Akbari, Zatollah Asemi, Reza Daneshvar Kakhaki, Fereshteh Bahmani, Ebrahim Kouchaki, Omid Reza Tamtaji, Gholam Ali Hamidi, Mahmoud Salami

**Affiliations:** ^1^Physiology Research Center, Kashan University of Medical SciencesKashan, Iran; ^2^Research Center for Biochemistry and Nutrition in Metabolic Diseases, Kashan University of Medical SciencesKashan, Iran; ^3^Department of Neurology, School of Medicine, Kashan University of Medical SciencesKashan, Iran

**Keywords:** Alzheimer's disease, clinical trial, cognitive function, metabolic status, probiotic

## Abstract

Alzheimer's disease (AD) is associated with severe cognitive impairments as well as some metabolic defects. Scant studies in animal models indicate a link between probiotics and cognitive function. This randomized, double-blind, and controlled clinical trial was conducted among 60 AD patients to assess the effects of probiotic supplementation on cognitive function and metabolic status. The patients were randomly divided into two groups (*n* = 30 in each group) treating with either milk (control group) or a mixture of probiotics (probiotic group). The probiotic supplemented group took 200 ml/day probiotic milk containing *Lactobacillus acidophilus, Lactobacillus casei, Bifidobacterium bifidum*, and *Lactobacillus fermentum* (2 × 10^9^ CFU/g for each) for 12 weeks. Mini-mental state examination (MMSE) score was recorded in all subjects before and after the treatment. Pre- and post-treatment fasting blood samples were obtained to determine the related markers. After 12 weeks intervention, compared with the control group (−5.03% ± 3.00), the probiotic treated (+27.90% ± 8.07) patients showed a significant improvement in the MMSE score (*P* <0.001). In addition, changes in plasma malondialdehyde (−22.01% ± 4.84 vs. +2.67% ± 3.86 μmol/L, *P* <0.001), serum high-sensitivity C-reactive protein (−17.61% ± 3.70 vs. +45.26% ± 3.50 μg/mL, *P* <0.001), homeostasis model of assessment-estimated insulin resistance (+28.84% ± 13.34 vs. +76.95% ± 24.60, *P* = 0.002), Beta cell function (+3.45% ± 10.91 vs. +75.62% ± 23.18, *P* = 0.001), serum triglycerides (−20.29% ± 4.49 vs. −0.16% ± 5.24 mg/dL, *P* = 0.003), and quantitative insulin sensitivity check index (−1.83 ± 1.26 vs. −4.66 ± 1.70, *P* = 0.006) in the probiotic group were significantly varied compared to the control group. We found that the probiotic treatment had no considerable effect on other biomarkers of oxidative stress and inflammation, fasting plasma glucose, and other lipid profiles. Overall, the current study demonstrated that probiotic consumption for 12 weeks positively affects cognitive function and some metabolic statuses in the AD patients. Clinical Trial Registration: http://www.irct.ir/, IRCT201511305623N60.

## Background

Alzheimer's disease (AD) is recognized as one of the most common forms of senile dementia (Qiu et al., 2007). AD begins with memory loss of recent events (short-term memory impairment) and finally robs the patients of their sense of self (Amemori et al., 2015). Early onset of the disease, older age, low education level, and several poor health conditions affect the prevalence rate of the disease and the degree of cognitive impairment (de Souza-Talarico et al., 2016). Increased biomarkers of oxidative stress (Furman et al., 2016), inflammation and chronic neuroinflammation are reported to be associated with many neurodegenerative disorders of central nervous system (CNS) including AD (Leszek et al., 2016). Furthermore, previous studies have demonstrated that metabolic alterations such as insulin resistance (Arrieta-Cruz and Gutierrez-Juarez, 2016), hyperglycemia (Arrieta-Cruz and Gutierrez-Juarez, 2016), and dyslipidemia (Presecki et al., 2011) are associated with the pathogenesis and development of AD. Experimental evidence indicates that alterations in micronutrients are also among the risk factors for AD (Taghizadeh et al., 2014).

The microbiota is a dynamic ecosystem which is influenced by several factors including genetics, diet, metabolism, age, geography, antibiotic treatment, and stress (Hufeldt et al., 2010; Cho et al., 2012; Drago et al., 2012). Recent evidence indicates a clear association between changes in the microbiota and cognitive behaviors (Gareau, 2014). In addition, animal studies imply on the necessity of an optimal function of what is known as the microbiome-gut-brain axis in the behavioral as well as electrophysiological aspects of brain action (Davari et al., 2013). There is a preliminary research on the effect of probiotics on the prognosis of cognition (Bhattacharjee and Lukiw, 2013). However, data on the effects of probiotics on improving cognitive disorders are scarce (Bhattacharjee and Lukiw, 2013; Davari et al., 2013). Gareau reported that intestinal dysbiosis in germ free animals (containing no microbiota), bacterial infection with an enteric pathogen and administration of probiotics can modulate cognitive behaviors including learning and memory (Gareau, 2014).

Some complications such as cognitive disorders, oxidative stress, neuroinflammation, insulin resistance, and altered lipid metabolism, which are observable in AD, are identified to be influenced by the gut flora as well as probiotics. However, no direct study has considered the gut microbiota manipulation in AD patients. Hence, this clinical trial was designed to assess if reinforcement of the intestinal microbiota via probiotic supplementation helps to improve cognitive and metabolic disorders in the AD patients.

## Materials and methods

### Trial design

This study was a 12-week randomized, double-blind, and controlled clinical trial.

### Participants

Participants included in this study were people with AD (60–95 years old) residing at the Golabchi (Kashan, Iran) and Sadeghyeh (Esfahan, Iran) Welfare Organizations between December 2015 and February 2016. The AD patients were diagnosed following the NINDS-ADRDA criteria (McKhann et al., 1984) and revised criteria from the National Institute on Aging-Alzheimer's Association (Jack et al., 2011). Patients with metabolic disorders, chronic infections and/or other clinically relevant disorders with exception of AD and consuming probiotic supplements within 6 weeks prior to the study, taking other forms of probiotics including probiotic yogurt, kefir, and other fermented foods were excluded.

### Ethics statements

This trial was performed in accordance with the Declaration of Helsinki. Informed consent was received from all patients before beginning the study. The research was approved by the ethics committee of Kashan University of Medical Sciences (KUMS) and was registered in the Iranian website for registration of clinical trials (http://www.irct.ir) IRCT201511305623N60.

### Study design

At the onset of the study, all subjects were matched for disease severity based on gender, BMI, and age. The participants were then randomly divided into two groups to receive either milk (control group, *n* = 30: 24 females and 6 males) or milk containing a mixture of probiotics (probiotic group, *n* = 30: 24 females and 6 males) for 12 weeks. The patients were requested not to change their ordinary physical activity and not to take any nutritional supplements during the 12-week trial. A trained researcher recorded dietary intakes (3-day food records) of all participants at the study baseline, the 3rd, 6th, and 9th week of the intervention and at the end of the trial. Daily macronutrient and micronutrient intakes were analyzed by nutritionist IV software (First Databank, San Bruno, CA). The nutritional questionnaire was completed by a trained researcher (the first author: E. Akbari).

### Intervention

In the intervention group (*n* = 30), patients received 200 ml/day probiotic milk containing *Lactobacillus acidophilus, Lactobacillus casei, Bifidobacterium bifidum*, and *Lactobacillus fermentum* (2 × 10^9^ CFU/g for each) for 12 weeks. It is generally agreed that probiotic strains should be of host origin with a beneficial effect on the host, withstand into food stuff with a high count, withstand transits through intestine and colonize lumen of the tract, produce antimicrobial agents, and technologically appropriate for industrial production (Shewale et al., 2014).

Due to the lack of evidence about the appropriate dosage of probiotics for AD, we used the above-mentioned doses based on few previous studies in healthy subjects (Benton et al., 2007; Mohammadi et al., 2015). Probiotic supplements were produced by Tak Gen Zist Pharmaceutical Company (Tehran, Iran) that was approved by the Food and Drug Organization of the Ministry of Health and Medical Education.

### Assessment of anthropometric measures

Weight and height of participants were determined in an overnight fasting status using a standard scale (Seca, Hamburg, Germany) at the onset of the study and after 12 weeks of the treatment. BMI was calculated as weight in kg divided by height in meters squared.

### Assessment of outcomes

The primary outcome measurements were Mini-Mental State Examination (MMSE) in the current study. The secondary outcome measurements were biomarkers of oxidative stress, inflammation and metabolic profiles. The MMSE was used to assess cognition in the AD subjects (Folstein et al., 1975).

### Assessment of biochemical parameters

Twelve-hour fasting blood samples were collected by venipuncture at weeks 0 and 12 at Kashan Reference Laboratory. The blood samples were taken according to a standard protocol and immediately centrifuged (Hettich D-78532, Tuttlingen, Germany). Then, the samples were stored at −80°C until analysis. Plasma total antioxidant capacity (TAC) was measured using the ferric reducing antioxidant power method developed by Benzie and Strain (1996). Total glutathione (GSH) was assessed by the method of Beutler et al. (Beutler and Gelbart, 1985). Malondialdehyde (MDA) concentrations was evaluated using the thiobarbituric acid reactive substance method (Janero, 1990). Serum high sensitivity C-reactive protein (hs-CRP) concentrations were quantified by the use of commercial ELISA kit (LDN, Nordhorn, Germany) with the intra- and inter-assay CVs 3.5 and 5.4%, respectively. Plasma nitric oxide (NO) were quantified by the Griess method (Tatsch et al., 2011). Available kits (Pars Azmun, Tehran, Iran) were used to determine the concentrations of fasting plasma glucose (FPG), serum triglyceride (TG), total holesterol, LDL, and HDL. All inter- and intra-assay CVs for NO, TAC, GSH, MDA, FPG, and lipid profiles were lower than 5%. Circulating levels of serum insulin were assessed using ELISA kit (Monobind, California, USA) with the intra- and inter-assay CVs 3.1 and 4.9%, respectively. The homeostatic model of assessment for insulin resistance (HOMA-IR), homeostatic model assessment for B-cell function (HOMA-B) and the quantitative insulin sensitivity check index (QUICKI) were calculated according to suggested formulas (Pisprasert et al., 2013).

### Statistical methods

To determine whether or not the study variables were normally distributed, we applied the Kolmogrov-Smirnov test to the data. Analyses were conducted based on an intention-to-treat (ITT) principle. Missing values were treated based on Last-Observation-Carried-Forward method (LOCF) (Lachin, 2016). LOCF ignores whether the participant's condition was improving or deteriorating at the time of dropout, instead, it freezes outcomes at the values observed before dropout (i.e., last observation) (Lachin, 2016). For non-normally distributed variables (FPG, insulin, HOMA-IR and hs-CRP), we applied Log transformation. Independent samples Student's *t*-test was used to detect the differences in anthropometric measures as well as in macronutrient and micronutrient intakes between the two groups. For comparison of the categorical variables, Pearson Chi-square test was used. To determine the effects of probiotic milk consumption on MMSE, biomarkers of oxidative stress, inflammation, and metabolic profiles, we used independent samples Student's *t*-test. Adjustment for changes in the baseline values of biochemical parameters, age and BMI at was performed by analysis of covariance (ANCOVA) using general linear models. The *P*-value of <0.05 were considered statistically significant. All statistical analyses used the Statistical Package for Social Science version 18 (SPSS Inc., Chicago, Illinois, USA). To calculate sample size, we used the standard formula suggested for clinical trials by considering type one error (α) of 0.05 and type two error (β) of 0.20 (power = 80%). Based on a previous study (Malaguarnera et al., 2007), we used 1.3 as SD and 1.1 as the difference in mean (d) of MMSE as key variable. Accordingly, we needed 25 persons in each group. Assuming 5 dropouts in each group, the final sample size was determined to be 30 persons per group. Randomization assignment was conducted using computer-generated random numbers. Randomization and allocation were concealed from the researchers and individuals until the final analyses were completed. The randomized allocation sequence, enrolling subjects and allocating them to interventions were conducted by a trained staff at the Welfare Organizations.

## Results

**Figure 1:**
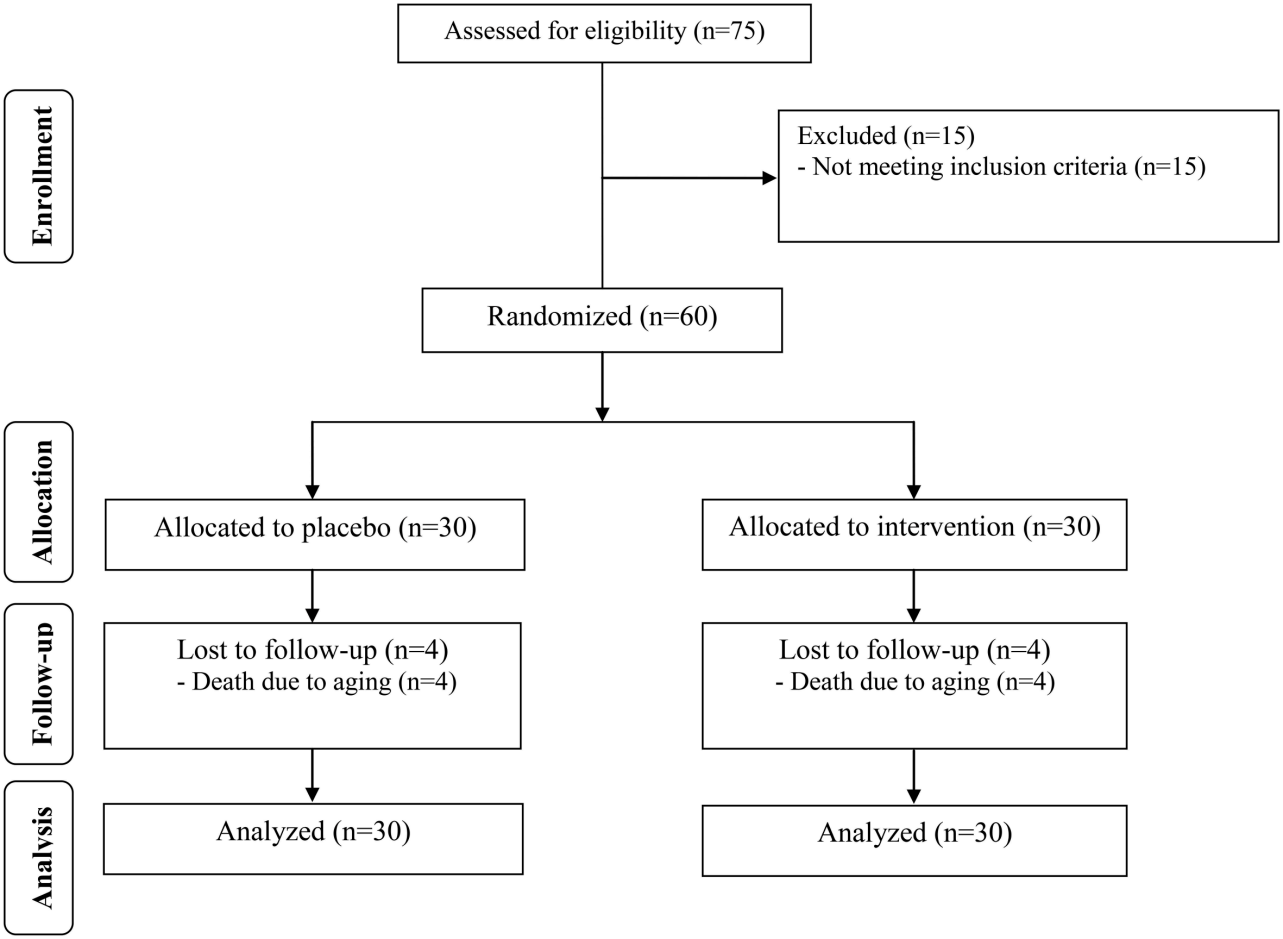
**Summary of patient flow**.

All participants were introduced to the MMSE cognitive test. Also the blood samples were assessed for the biomarkers status. During the study 4 out of 30 patients in the two groups were died and a total of 52 subjects [control (*n* = 26) and probiotic (*n* = 26)] completed the experiments (Figure 1). However, as the analysis was based on the ITT principle, all 60 patients were included in the final analysis. The between group comparisons are made considering the post-treatment changes in the baseline values.

No side effects were reported following administration with probiotic in AD patients throughout the study. Mean age, height, weight, and BMI at baseline and end of trial were not statistically different between the two groups (Table 1). Based on the 3-day dietary records obtained at study baseline, end of trial and throughout the trial, we found no significant difference in mean dietary macronutrient and micronutrient intakes between the two groups (Data not shown).

**Table 1:** **General characteristics of the participants (firstly entered the study)**.

	**Control group (*n* = 30)**	**Probiotic group (*n* = 30)**	**P^a^**
**GENDER (%)**			
Male	6 (20.0)	6 (20.0)	1.00^†^
Female	24 (80.0)	24 (80.0)	
Age (y)	82.00±1.69	77.67±2.62	0.13
Height (cm)	157.43±1.86	157.77±2.03	0.90
Weight at study baseline (kg)	56.63±2.21	59.03±1.99	0.42
Weight at end-of-trial (kg)	56.80±2.17	59.50±1.98	0.36
Weight change (%)	0.37±0.41	0.85±0.27	0.25
BMI at study baseline (kg/m^2^)	22.73±0.68	23.77±0.73	0.30
BMI at end-of-trial (kg/m^2^)	22.81±0.67	23.95±0.72	0.25
BMI change (%)	0.37±0.41	0.85±0.27	0.31

*Data are mean ± SEM*.

a *P-values obtained from independent t-test*.

† *Obtained from Pearson Chi-square test*.

### Cognitive assessment

The degree of cognitive impairments in the probiotic and control groups was evaluated by the MMSE test. Twelve weeks intervention resulted in an improvement in MMSE score in the probiotic group (+27.90% ± 8.07) compared to their control counterparts (−5.03% ± 3.00). The difference between the two groups of testing was statistically significant (*P* <0.001).

### Biochemical measurements

Twelve weeks probiotic treatment decreased the level of the factors affecting metabolism of carbohydrates. The changes in hs-CRP were −17.61% ± 3.70 and +45.26% ± 3.50 μg/ml in the probiotic and control groups, respectively (*P* <0.001). HOMH-IR index decreased in the probiotic group (+28.84% ± 13.34) compared to their control counterparts (+76.95% ± 24.60, *P* = 0.002). The probiotic treatment considerably reduced the HOMA-B index (+3.45% ± 10.91) in the probiotic compared to the control (+75.62% ± 23.18) patients leading to a significant variation between the two groups (*P* = 0.001). The QUICKI level was significantly (*P* = 0.006) increased in the probiotic subjects (−1.83% ± 1.26) in comparison to their control counterparts (−4.66% ± 1.70). On the other hand, the probiotic supplementation was ineffective on the FBP; the changes are (3.41% ± 2.12) and (4.50% ± 4.39) mg/dl in the probiotic and control groups, respectively.

The probiotic supplementation differently influenced the lipid profiles. The TG level was substantially decreased (*P* = 0.003) in the probiotic patients (−20.29% ± 4.49 mg/dl) compared to the control group (−0.16% ± 5.24 mg/dl). Although the concentration of VLDL was reduced in the probiotic subjects compared to their control counterparts (−20.29% ± 4.49 vs. −0.16% ± 5.24 mg/dl, *P* = 0.003), however, the other lipid profiles (LDL, HDL and cholesterol) were insensitive to the probiotic treatment.

It should be noted that the baseline levels of serum triglycerides, VLDL, and HDL were significantly different between the two groups. Therefore, we controlled the analyses for the baseline values of biochemical parameters, age, and baseline BMI. Nevertheless, this adjustment indicated no considerable changes in our findings (Data not shown).

The probiotic treatment caused a significant decrease (*P* <0.001) in the MDA of the probiotic group (−22.01% ± 4.84 μmol/l) in comparison to the control patients (+2.67% ± 3.86 μmol/l). We found no difference in the level of the TAC and NO between the two groups.

Table 2 explains the pre- and post-trial values in the control and probiotic patients.

**Table 2:** **Mean values of the behavioral test and the biomarkers measurements in the probiotic and control groups**.

	**Control group**		**Probiotic group**		**Difference between the two groups**
	**Baseline**	**End-of-trial**	**Baseline**	**End-of-trial**	***P*-value^a^**
MMSE (score out of 30)	8.47±1.10	8.00±1.08	8.67±1.44	10.57±1.64	<0.001
TAC (mmol/L)	895.66±25.96	915.35±26.60	876.13±26.48	922.42±28.53	0.25
GSH (μmol/L)	390.78±17.46	386.76±20.33	377.26±14.82	401.25±16.68	0.19
MDA (μmol/L)	4.26±0.30	4.32±0.31	4.31±0.26	3.21±0.23	<0.001
hs-CRP (μg/ml)	4.54±1.30	6.59±1.14	6.61±1.24	5.44±0.85	<0.001
NO (ìmol/L)	44.76±0.53	45.56±0.82	43.68±0.64	44.37±1.14	0.93
FPG (mg/dl)	83.40±2.36	86.77±4.07	92.00±7.92	94.13±7.72	0.98
HOMA-IR	1.43±0.24	2.08±0.27	1.30±0.13	1.60±0.19	0.002
HOMA-B	25.04±3.21	37.86±4.64	27.36±3.50	22.06±2.43	0.001
QUICKI	0.38±0.01	0.36±0.01	0.38±0.01	0.37±0.01	0.006
Triglycerides (mg/dl)	84.32±4.65	81.74±4.76	119.60±10.25	94.33±10.04	0.003
VLDL (mg/dL)	16.86±0.93	16.35±0.95	23.92±2.05	18.87±2.01	0.003
LDL (mg/dl)	90.44±4.58	94.34±4.39	85.16±4.14	90.64±5.29	0.76
HDL (mg/dl)	51.27±1.75	44.49±1.97	45.81±1.45	38.82±1.35	0.93
Total cholesterol (mg/dl)	158.57±5.75	155.17±5.59	154.88±4.91	148.32±5.43	0.63
Total/ HDL-cholesterol	3.15±0.12	3.62±0.16	3.43±0.12	3.95±0.2	0.81

*Data are mean ± SEM*.

a *represents P-values obtained from the time × group interaction analysis. FPG, fasting plasma glucose; GSH, total glutathione; HOMA-IR, homeostasis model of assessment-estimated insulin resistance; HOMA-B, homeostasis model of assessment-estimated B cell function; hs-CRP, high-sensitivity C-reactive protein; MMSE, mini-mental state examination; MDA, malondialdehyde; NO, nitric oxide; QUICKI, quantitative insulin sensitivity check index; TAC, total antioxidant capacity*.

## Discussion

The current study demonstrated that the probiotic administration for 12 weeks has favorable effects on MMSE score, MDA, hs-CRP, markers of insulin metabolism and triglycerides levels of the AD patients; however, the changes in other biomarkers of oxidative stress and inflammation, FPG and other lipid profiles are negligible. To the best of our knowledge, this study is the first evaluating the beneficial effects of probiotic supplementation on cognitive function, biomarkers of oxidative stress, inflammation and metabolic status in patients with AD. AD patients are predisposed to some complications including increased oxidative stress (Sultana et al., 2011), morbidity, mortality (Schelke et al., 2016), microvascular disease, dyslipidemia, and insulin resistance (Sridhar et al., 2015). Our results indicated that the probiotic treated patients showed some improvement in their MMSE scores. Studies considering the effect of probiotic supplements on brain behavioral phenomena are scant. In our previous work, we have shown that probiotics efficiently reverse the impaired spatial learning and memory as well as synaptic transmission in diabetes mellitus (Davari et al., 2013). It is demonstrated that other brain related disorders such as multiple sclerosis (Kouchaki et al., 2016) and stress are also influenced by probiotics (Liang et al., 2015). In the levels of molecular mechanism, microbiome is known to play a pronounced role in synaptic transmission. Numerous studies have shown capability of bacteria in producing neurotransmitters and neuromodulators including gamma-aminobutyric acid (GABA), norepinephrine, serotonin, dopamine, and acetylcholine (Cryan and Dinan, 2012). Further, findings from germ free animals indicated a decreased level of brain derived neurotrophic factor (BDNF), important neurotrophic factor in the neuronal growth and survival, and a reduced expression of some subunits of *N*-Methyl-D-aspartate (NMDA) receptors (Sudo et al., 2004) involved in most abundant neurotransmission in brain (Salami et al., 2000). GABA is the major inhibitory neurotransmitter in the CNS. Dysfunctions in GABA-signaling are linked to anxiety and depression, defects in synaptogenesis and cognitive impairments (Aziz et al., 2013; Mitew et al., 2013). Also the glutamatergic NMDA receptors are involved in the most important excitatory neurotransmission of brain (Talaei et al., 2016) which is engaged in the neural circuits involved in learning and memory. From these considerations it can be concluded that, at least through contributing in neurotransmitter synthesis or receptor expression, probiotics might adjust the brain activity.

Findings of the current study indicated that while the probiotic supplementation decreased the plasma MDA and the serum hs-CRP levels it was ineffective on other biomarkers of oxidative stress and inflammation. Consistent to our findings, a significant reduction in MDA levels was reported in type 2 diabetic patients after consuming probiotic yogurt (Ejtahed et al., 2012). In addition, Zarrati et al. (2014) showed that 8 weeks consumption of yogurt, enriched by *L. acidophilus, Bifidobacterium langum*, and *L. casei* (10^8^ CFU/g each) decreased inflammatory cytokines in overweight people. In contrast, others reported no beneficial effects of probiotics on biomarkers of oxidative stress and inflammation. For example, it was reported that 6 weeks probiotic treatment caused no significant change in inflammatory factors of diabetic patients (Mazloom et al., 2013). Furthermore, 8 weeks consumption of capsules containing 10^8^ CFU/g of *L. casei* by the people suffered from rheumatoid arthritis displayed no significant within- and between-group changes in MDA and TAC levels (Vaghef-Mehrabany et al., 2015). Oxidative stress is a frequently observed feature of AD, although its pathological significance is not understood (Selvarajah et al., 2011). Evidence indicates that, through production of reactive oxygen species, oxidative stress acts as vehicle for deposition and accumulation of amyloid β (Aβ) in AD (Kim et al., 2015). It has also been postulated that oxidative stress may decrease the activity of α-secretase, which promotes the expression and activity of β and γ-secretase, leading to the increased production of Aβ (Tan et al., 2013). It seems that probable anti-inflammatory role of probiotics needs further investigation to be cleared. Altogether, since the microbiota-gut-brain axis conduits some of its main actions through nerve pathways both dysbiosis and probiotic treatment could have profound effects on the CNS functions. Accumulating evidence from experimental studies supports the hypothesis that; via affecting inflammation, endocrine system, and neurotransmission; the gut microbiome takes a crucial role in the CNS function (Collins et al., 2012; Dinan and Cryan, 2013). Accordingly, it is suggested that dysfunction of the neuroendocrine system, behavior, and cognition are correlated with gut microbiota dysbiosis (Liang et al., 2015). These considerations led to establishing the term “psychobiotics” to highlight the potential effects of probiotics in treatment of mental disorders (Wall et al., 2014). Consistently, in a meta-analysis study, Kasińska and Drzewoski (2015) reported a reduced HOMA-IR and insignificant FGP in probiotic treated subjects. Mazloom et al. (2013) also reported that probiotic supplementation had no significant effect on fasting blood glucose, markers of insulin metabolism and lipid profiles. Consumption of symbiotic bread containing the heat-resistant probiotic *Lactobacillus sporogenes* (1 × 10^8^ CFU/g) for 8 weeks also decreased the serum triglyceride and VLDL concentrations in patients with type 2 diabetes (Shakeri et al., 2014).

Emerging evidence has demonstrated that brain insulin resistance, as a key mediator in prediabetes and diabetes mellitus, may take a role in AD (Cervellati et al., 2016). Insulin resistance plays an important role in the development of cognitive impairment in primary elderly hypertensive patients (Ma et al., 2015). On the other hand, the role of lipids in the etiology and progress of AD is still unclear. Some evidence from clinical studies support the fact that abnormal cholesterol metabolism in the brain leads to progressive cognitive dysfunction (Wang et al., 2014). Accordingly, high lipid levels could be one of the risk factors for AD (Reitz et al., 2010). Probiotics intake may improve markers of insulin metabolism and lipid profiles by reducing cytokines and suppressing the nuclear factor kappa-light-chain-enhancer of activated B cells (NF-κB) pathway (Shi et al., 2006) and gut microbiota-short chain fatty acids (SCFA)-hormone axis (Yadav et al., 2013).

The current study had some limitations. Measurement of fecal bacteria loads before and after the probiotic supplementation in the AD patients was very difficult. In addition, we assessed cognition of the AD patients based on only MMSE test. Hence, considering some other cognitive criteria could be helpful in confirming relevancy of cognition to probiotic supplementation. Also evaluation of other biomarkers of inflammation and oxidative stress including interleukin 6 (IL-6), tumor necrosis factor alpha (TNF-α), catalase and superoxide dismutase (SOD) seems to be informative.

Overall, the current study demonstrated that 12 weeks consumption of probiotic in the AD patients had favorable effects on MDA, hs-CRP, markers of insulin metabolism, and serum levels of triglyceride and VLDL. However, the pobiotic treatment was inefective on other biomarkers of oxidative stress and inflammation, FPG, and other lipid profiles. Considering the MMSE data we concluded that the probiotic supplementation shows some hopeful trends that warrant further study to assess if probiotics have a clinically significant impact on the cognitive symptoms.

## Clinical registration

http://www.irct.ir: IRCT201511305623N60.

## Author contributions

MS designed the research project. EA had principal role in performing the protocol. RD visited all patients for the MMSE tests. EK, GAH and OT assisted in performing the protocol. ZA and FB performed measurement of metabolic biomarkers. ZA analyzed the data. The manuscript was written by MS and ZA. Final edit was accomplished by MS.

### Conflict of interest statement

The authors declare that the research was conducted in the absence of any commercial or financial relationships that could be construed as a potential conflict of interest.
