# Expression of Concern: Effect of Probiotic Supplementation on Cognitive Function and Metabolic Status in Alzheimer's Disease: A Randomized, Double-Blind and Controlled Trial

**DOI:** 10.3389/fnagi.2020.602204

**Published:** 2020-10-07

**Authors:** Frontiers Editorial Office

With this notice, Frontiers states its awareness of concerns regarding the validity of the participant data in this study. An investigation is currently being conducted by Kashan University of Medical Sciences research ethics committee. This expression of concern has been posted while Frontiers awaits the outcome of that investigation and will then be updated accordingly.

